# 

**Published:** 1900-01

**Authors:** 


### ILLUSTRATIONS.

PAGE

Carpipes Unilateralis Congenitalis et Luxatio Capiti Radii (Congenitalis) . . . . . . . . . . . . 544, 545

Dawson, French Canine Hopples . . . . . . . . . 629

Kenney, Richard R., United States Senator from Delaware . . 518

Pneumonomycosis . . . . . . . . . 458, 459, 462, 463

Urinary Analysis . . . . . . . . . . . . 610

### PERSONALS.

Dr E. Newcomer, of Mt. Joy, Pa., was a visitor to Philadelphia in the early part of December.

Among the leading candidates for the position of State Veterinarian in Maryland are Dr. Clement, for reappointment; Dr. Robert Ward, who preceded the present incumbent ; Drs. Grogan and Martinet, all of Baltimore, and Dr. F. H. Mackie, of Fair-

Hill, member of the State Board of Veterinary Medical Examiners. The race promises to be a heated one, and several dark horses are under special grooming for the much-coveted position. The joys of public place are full of anxious ones in attainment.

Dr. J. D. Bell, of Flushing, Michigan, has succeeded by purchase to the practice of Dr. A. L. Compton, at Williamston.

Dr. S. W. McClure, of Oxford, Pa., was a visitor to Philadelphia in December after a ten days' sojourn in North Carolina, following the trophies of the gun.

Dr. R. S. Huidekoper returned to Washington early in December to again assist in the directing of efforts looking forward to the attainment of better recognition of the veterinarian in the United States Army.

Dr. Leonard Pearson, State Veterinarian of Pennsylvania, was a visitor to the city of Pittsburg early in December on official business.

Dr. A. L. Compton, of Williamston, has gone to Morrice, Michigan, where he has engaged in practice.

Dr. Charles M. Frantz, of Lehighton, is now a resident of Philadelphia, but is not engaged in veterinary practice.

Dr. C. E. Tomlinson, formerly of Williamsport, is now a resident of Panther, West Virginia, having entered upon a professional contract with one of the largest lumber merchants of that territory. His duties are mostly performed on horseback, and cover many miles among the lumber camps.

Dr. W. H. Ridge, of Trevose, Pa., takes an active part in directing the affairs of the Southampton Farmers' Club.

Dr. W. N. D. Bird, recently located at Nashville under the Bureau of Animal Industry, has been transferred to St. Louis, Mo.

Dr. G. R. Neson, State Veterinarian of South Carolina, will shortly issue a bulletin on tuberculosis, showing the result of some two hundred tests. One herd of sixty, supplying milk to a large number of Charleston families, revealed the presence of tuberculosis in thirty-one head of these animals. The sale of a portion of these animals was followed by the owner resuming his business, and in the absence of specific laws he suffered only a temporary decrease in the sale of the product.

Dr. Louis A. Klein, of Philadelphia, will lecture on "Meat Inspection" at the Veterinary Department of the University of Pennsylvania the remaining portion of the college year.

Prof. Wesley Mills, of Montreal, Canada, has gone to Leipsic, Germany, for a period.

Dr. Joseph Plaskett, of Nashville, Tenn., successfully passed the civil-service examination, and on the eve of a command to report for duty at Chicago was selected by the English Government to accompany a shipment of mules by transport to South Africa.

Dr. Daniel Lemay, of the U. S. Army, is now located with the 7th Cavalry at Columbia Barracks, Havana, Cuba.

Dr. E. A. A. Grange, now of New York City, has an interesting article in the December 30th issue of the *Rider and Driver*, on " The Knights of the Leather Apron and Iron Horseshoe."

Dr. Charles A. Dohan, of Darling, Pa., is master of the hounds of the Lima Hunt Club, and daily leads the lovers of this sport in their following of the hounds.

Dr. G. W. Dunphy, State Veterinarian of Michigan, recently discussed the milk-question and the danger of the transmission of tuberculosis before the Fourth Annual Conference of Health Officials of the " Wolverine State."

Dr. Wm. Dougherty, of Baltimore, Md., entertained a number of his male friends of that city on Christmas day in a thoroughly royal manner.

Dr. Harry Marshall, of Georgetown, Del., was a visitor to Philadelphia in the latter part of November.

Mr. R. A. Pearson, of the Dairy Division of the Department of Agriculture, was a Thanksgiving-day visitor to the " City of Brotherly Love." He never loses an opportunity of focussing types of dairy stock, and makes good use of his pastime with the camera.

Dr. Leonard Pearson, State Veterinarian of Pennsylvania, addressed the Southampton Farmers' Institute on January 9th on " The Relation of Stabling Conditions to the Spread of Tuberculosis."

Dr. A. W. Ormiston, of Germantown, Pa., is an enthusiastic breeder of beagle hounds, and their sharp, shrill voices are the sweetest music to his ears in the gunning season.

Dr. S. L. Blount, who recently located at Martinsville, West Virginia, has been engaged by British officials to accompany an export of some nine hundred mules to South Africa.

